# Examinations by State Board, Atlanta, June 29

**Published:** 1897-08

**Authors:** 


### EXAMINATIONS BY STATE BOARD, ATLANTA, JUNE 29.

ANATOMY.—Dr. F. M. Ridley.

1. Name bones of head? (*a*) Cranial. (*b*) Facial.

2. Name bones of trunk?

3. (*a*) Name bones of one upper extremity? (*b*) Name bones of one lower extremity? (*c*) Name unclassified bones?

4. (*a*) What are arteries? (*b*) Name branches of arch of Aorta? (*c*) What arteries supply tissues of heart?

5. Name divisions in order of (*a*) Small Intestine, (*b*) Large Intestine?

### SURGERY.—Dr. F. M. Ridley.

1. Define Fracture? Give differential diagnosis between fracture and dislocation?

2. Give differential diagnosis between cerebral concussion and compression. Briefly give rational treatment of each.

3. What is aneurism? What do you understand by dissecting aneurism? What is differential diagnosis between abscess and aneurism?

4. What is Talipes Varus? Valgus?

5. Describe and give treatment of fistula in ano? Give approved treatment for hemorrhoids?

### GYNECOLOGY.—Dr. J. B. S. Holmes.

1. Give symptoms and treatment of acute pelvic Peritonitis?

2. Define Salpingitis and how would you treat it?

3. What is Rectocele, Urethrocele and Cystocele? How should they be treated?

4. How would you diagnose and treat Carcinoma Uteri?

5. How would you diagnose and treat uterine Myo-fibroma?

### OBSTETRICS.—Dr. J. B. S. Holmes.

1. Mention the internal organs of generation of the female and briefly describe the uterus?

2. What do you understand by Ectopic Gestation and how would you recognize and treat it?

3. How would you treat an inevitable abortion?

4. What is the best method of delivering the placenta?

5. What are the ordinary duties of a physician in a case of labor?

### CHEMISTRY.—Dr. E. R. Anthony.

1. What five elements are gases in their natural state?

2. What is the difference between a mixture and a compound?

3. Why is the Chlorine group called Halogens?

4. Name a Hydrogen Acid and give its formula? Also an Oxygen Acid and give its formula?

5. What is the clinical significance of Indican in the Urine? Give a good test for same.

### PHYSIOLOGY.—Dr. E. R. Anthony.

1. Name the varieties of Epithelium tissue, and state where found.

2. Name the different kinds of glands found in the stomach and give their functions.

3. What causes the pulse? State what conditions other than disease modify the pulse.

4. About what is the size of an air cell, and about how many constitute a lobule?

5. Define a nerve, a plexus, a commissure and a decussation.

### PRACTICE.—Dr. A. A. Smith.

1. Give prominent symptoms and treatment of pleurisy.

2. Give symptoms of Pericarditis, also treatment for same.

3. Give treatment of Spasmodic Croup.

4. Give definition, symptoms and treatment for Acute Gastritis.

5. What is Varicella?

6. Give differential diagnosis features in Variola and Varicella.

7. Give symptoms and treatment of Diphtheria.

8. Name some of the principal eruptive diseases.

### MATERIA MEDICA AND THERAPEUTICS.—Dr. W. A. O'Daniel.

1. Describe the methods of Massage and its therapeutic uses.

2. Give the most important therapeutic uses of water.

3. Give the modes of applying heat and cold and the physiological action of each.

4. Do acids produce "salivation" in a patient taking the various preparations of mercury? If so, why so?

5. Give the dose and physiological action of Liquor Potassii Arsenitis? Also the antidote for poisoning by arsenic?

6. How would you prepare Quiniæ-Sulphas for hypodermic medication? Write me a prescription for same.

7. Describe the difference between acute alcoholism and compression of the brain?

8. Give the dose, physiological action and therapeutic uses of the official preparations of Hydrastis Canadensis? Also its antagonistics and incompatibles?

---

Our readers who have read of the serious illness of Dr. Virgil O. Hardon, of this city, will be pleased to learn that he is rapidly recovering. In the last few years Dr. Hardon has had three mild attacks of appendicitis and about July 2d, last, he was seized with another, more severe than any of the preceding. An operation was imperatively demanded and this was performed July 4th by Drs. McRae, Cooper and Elkin. For several days it was hardly expected that the patient would survive. From the third day, however, improvement began and has steadily continued. Dr. Hardon will spend the month of August recuperating in the North, and will return to Atlanta September 1st.

